# Factors related to lower limb performance in children and adolescents aged 7 to 17 years: A systematic review with meta-analysis

**DOI:** 10.1371/journal.pone.0258144

**Published:** 2021-10-06

**Authors:** Paulo Francisco de Almeida-Neto, Vitória Monteiro Monte Oliveira, Dihogo Gama de Matos, Ísis Kelly dos Santos, Adam Baxter-Jones, Vanessa Carla Monteiro Pinto, Tatianny de Macêdo Cesário, Felipe J. Aidar, Paulo Moreira Silva Dantas, Breno Guilherme de Araújo Tinôco Cabral

**Affiliations:** 1 Health Sciences Center, Department of Physical Education, Federal University of Rio Grande do Norte, DEF-UFRN, Natal, RN, Brazil; 2 Department of Physical Education—State University of Ceará, UECE, Fortaleza, Brazil; 3 Cardiovascular & Physiology of Exercise Laboratory, Faculty of Kinesiology and Recreation Management, University of Manitoba, Winnipeg, Canada; 4 Post Graduate Program in Health Sciences, Federal University of Rio Grande do Norte, Natal, RN, Brazil; 5 College of Kinesiology, University of Saskatchewan, Saskatoon, SK, Canada; 6 Department of Physical Education, Federal University of Sergipe, UFS, São Cristovão, SE, Brazil; The Wingate College of Physical Education and Sports Sciences at the Wingate Institute, ISRAEL

## Abstract

**Background:**

The literature identifies several factors that are associated with lower limb performance (LLP). However, there is little consensus on which factors have the major associations with LLP.

**Objective:**

Examine, analyze and summarize the scientific evidence on the factors associated with the performance of LLP in children and adolescents of both sexes aged between 7 and 17 years.

**Design:**

This systematic review was conducted following the Preferred Reporting Items for Systematic Review and Meta-Analysis (PRISMA) statement and was registered in PROSPERO.

**Data sources:**

A systematic literature search of five electronic databases (i.e., SPORTDiscus, PubMed, CINAHL, Google Scholar, and SCOPUS) with date restrictions was conducted (2010 to 2021).

**Eligibility criteria for selecting studies:**

Eligibility criteria included (i) a study published between 2010 and 2021; (ii) a research study with observational design; (iii) a study analyzing LLP; and (iv) a sample composed of young people between 7 and 17 years old (regardless of sex).

**Analyses:**

Literature analysis was carried out in English and Portuguese between 2018 and 2021, “blindly” by two researchers. For data sorting, Rayyan® was used. Data extraction and evidence analysis were performed “blindly”, using the Loney scale. The minimum items for observational studies were analyzed by the STROBE checklist. Meta-analyses were conducted based on age group (Childhood [7 to 11 Yrs] and Adolescence [12 to 17 Yrs]) and puberty stages (i.e., Prepupertal and Pubertal). The heterogeneity between the samples of the studies was assessed using the “Cochran’s Q” and “I^2” statistics. Meta-regression analyses were performed to check the factors related to heterogeneity of the studies and to check the associations between chronological age and LLP.

**Results:**

The literature search resulted in 1,109,650 observational studies of which 39 were included in this review. Through Meta-analysis and Meta-regressions, it was possible to indicate that advancing chronological age related to increased LLP (p<0.01), and that in relation to puberty stages pubertal subjects had higher LLP than their pre-pubertal peers (p<0.01).

**Discussion:**

The main findings of the present systematic review suggest that as chronological age advances (childhood to adolescence), neuromuscular systems mature and this may be due to advancing puberty, which is also associated with an increase in LLP.

**Conclusion:**

The factors associated with lower limbs performance are still inconsistent in the literature. However, advancing chronological age and stage of puberty are both associated with increased lower limbs performance.

**Trial registration:**

ID-PROSPERO-CRD42020137925.

## 1. Introduction

For children and adults, lower limbs performance (LLP) is considered a significant component for high performance in several sports (e.g,basketball, volleyball, football, karate, etc.) [1–4]. In addition, LLP is seen as an extremely important factor in determining physical fitness (i.e., competence in performing muscle work in a way that does not require high effort), and contributes to the performance of daily activities such as running, walking, sitting, and being able to get-up [5–7]. During childhood and adolescence, because of these relationships, LLP can be used as an indicator of motor performance and functional health (locomotors functions) [8, 9].

Lower limbs performance can be asssed using different protocols, among the most commonly used methods are analysis by jumping protocols and measurement by isokinetic dynamometers [10, 11]. Thus, using specific tests for LLP it is possible to classify the physical fitness of young people in relation to functionality and sports performance [10, 11]. Such results can contribute to guiding coaches and health professionals on appropriate modifications of training regimes to improve the health and motor performance of children and adolescents [10, 11]. It is noteworthy that, during puberty, the variability in differences in physical and motor aspects between individuals of the same chronological age (CA) can be very significant [12]. Given this assumption, when analysing LLP in young people it is necessary to take into account not just their chronological age but also their stage of biological maturation (BM) (i.e., improvement in biological functions) [12, 13].

During maturation, the neuromuscular system improves, this can result in young people with advanced puberty having higher levels of LLP compared to their CA matched. less advanced pubertaly, peers [12–14]. It should be noted that during puberty, young people have lower LLP levels in relation to the muscular performance of other body segments (i.e., upper limbs and trunk) [11, 15, 16]. One of the justifications is that during puberty, the maturation process follows a linear pattern of caudal-skull development [17].

Scheffler and Hermanussen [18], found that the neuromuscular system and morphological development are improved primarily in the upper segment of the human body, which may disadvantage the LLP compared to other body segments. Others, such as Silva and Oliveira [19], found no relationship between puberty and LLP levels. Physical activity is another factor that has been shown to have a relationship with LLP. Rover et al., [20] and Martins et al., [21] found that LLP was influenced by the level of physical activity, and may also be influenced by anatomical patterns such as the length of the lower limbs and statural height. Moreover, several authors in observational studies identify differences both favouring and not favouring LLP, such as advanced CA, being male, having predominantly a lean body morphology or having high levels of steroid hormones (i.e., testosterone and estradiol) [3, 5, 22–25]. More evidence is required to test the influence of biological maturation, as well as the associated factors, on LLP development. Such studies are required to ensure consideration of new approaches that contribute to better understanding of the independent role of maturation.

Hanlon et al., [26], identified that sports programs related to injury prevention in the lower limbs region of young athletes were are able to improve intrinsic factors, such as muscle strength, that are associated with motor coordination, posture and body balance. However, these authors did not investigate the same factors related to the increase in performance of LLP.

Currently there is no consensus in the literature as to what factors relate to LLP development. To the best of our knowledge, no systematic review has been conducted focusing on interventions related to factors that may influence the levels of LLP in children and adolescents. Therefore, it is necessary to systematize the current knowledge so that coaches and health professionals are able to explain which factors can be taken into account in order to improve LLP development in children and adolescents.

The objective of this systematic review was examine, analyse and summarize the scientific evidence of the factors associated with the LLP development in children and adolescents of both sexes, aged between 7 and 17 years.

## 2. Methods

### 2.1 Protocol and registration

This review adheres to the Preferred Reporting Items for Systematic Reviews and Meta-Analyses (PRISMA) statement for reporting systematic reviews and meta-analyses [27], according to the guidelines of the EQUATOR Reports (Improving the Quality and Transparency of Health Research). It was registered with the International Prospective Register of Systematic Reviews (PROSPERO; *ID*: n° CRD42020137925). In addition, this review strictly complied with all the criteria required by the AMSTAR check list (i.e., tool developed to judge the methodological quality of systematic reviews) [28].

### 2.2 Identification of studies and search strategy

In order to systematically define the initial details of the research strategy for the production of this review, the scheme described by the Joanna Briggs Institute was adopted, where the structure helps in defining the steps to be followed for the construction of the systematic review [29]. The method recommends the prior definition of the study population, the study interventions and the design of studies that are part of the review [29]. Thus, the population of the present study was defined as young people aged between 7 and 17 years old; the intervention considered was the evaluation of the LLP and the types of studies considered were observational.

For searches of academic articles in the databases, descriptors registered on the DEC’s and MeSH platforms were used primarily, the descriptors were connected through the Boolean operators OR / AND. It should be noted that the usual terms referring to the descriptors were also connected during the searches. Thus, the search strategy used was: Child OR Children AND Adolescent OR Adolescents OR Teenagers AND Performance AND Potency Muscle AND Explosive force AND Muscle Power. The searches were performed with the same word grid translated into Portuguese.

The aforementioned search strategy word grid was used in four phases of the present study (2nd semester 2018, 1st semester 2019, 2nd semester 2019, 1st semester 2020, details in the results section). However, the results were not satisfactory, which led the research team to review the methodology of the present study. After a thorough review, the word grid of the search strategy underwent changes in the Boolean operators: Child AND Children OR Adolescent AND Adolescents AND Teenagers OR Performance AND Potency Muscle OR Explosive force OR Muscle Power. The searches were performed with the same grid of words translated into Portuguese. The updated word grid in the search strategy was used in two more stages of the study (2nd semester 2020, 1st semester 2021, details in the results section), and the results were satisfactory.

### 2.3 Eligibility of the studies criteria

The evaluation of the eligibility of the studies was carried out independently by two researchers (P.F.A-N. and V.M.M.O.), using the electronic research platforms: PubMed, CINAHL, SPORT Discus, Google Scholar and SCOPUS (when available, the advanced search feature was used), between January 2018 and February 2021. The searches were made in both English and Portuguese. Intitaly, studies were considered for inclusion based on titles and abstracts. This procedure was organized in three separate steps: the first aimed to search the digital databases, while the second and third were aimed at identifying new publications. For the selection of works, the following eligibility criteria were adopted: (i) a study published between 2010 and 2021; (ii) a research study with observational design; (iii) a study analysing LLP; and (iv) a sample composed of young people between 7 and 17 years old (regardless of sex).

### 2.4 Data extraction

Data sorting occurred with the aid of the online tool Rayyan QCRI^®^ [30]. Rayyan QCRI^®^ is an open source application that allows the author, collaborators and research supervisor to have access to the systematic review data, ensuring the blindness of researchers during searches and data extraction [30]. The extraction of data and analysis of the quality of the evidence, were carried out “blindly” by two different researchers (P.F.A-N. and V.M.M.O.), each performed the recording of the main data from the studies. Subsequently, all researchers gathered the main results of the articles included in this review; it should be noted that only the data referring to LLP were maintained after data extraction.

### 2.5 Criteria for risk of bias assessment

The risk assessment of biases was carried out in a “blind” way, by three different researchers (I.K.S., P.M.S.D. and B.G.A.T.C.), using the quality scale of observational studies proposed by Loney et al. [31]. The final results of the evaluations were optimized and discussed by all the researchers involved (P.F.A-N., V.M.M.O., D.G.M., I.K.S., A.B-J., V.C.M.P, T.M.C., P.M.S.D., F.J.A. and B.G.A.T.C.). Methodological biases were analysed; results and reproducibility. The Loney scale considers the following points [31]: 1) The study does not answer the research question adequately. 2) The sample base and the sample size were not adequate. 3) Objective, adequate and standardized criteria for the outcome were not used. 4) The outcome was measured in a skewed manner. 5) Results are not given with a confidence interval when appropriate. 6) The study does not expose practical applicability. 7) The study has no reproducibility for other samples from different populations.

### 2.6 Analysis of the basic requirements that must be part of an observational study

It was verified whether the studies contained in the sample met the requirements required by STROBE (i.e., List of minimum items that must be integrated into observational studies) [32].

### 2.7 Analyses and synthesis of results

The effects of interventions that compared LLP among young people of different maturation stages [Pre-pubertal x Pubertal] and different age groups [7 to 17 years] were measured by the difference in standardized means and grouped using random effect models. The heterogeneity between the studies was assessed using the “Cochran’s Q” and “I^2” statistics. Studies were considered heterogeneous when: I^2> 50% [33]. Subgroup meta-analysis (considering sample sex, LLP assessment protocol and biological maturation assessment protocol)] and meta-regression (considering sample size, chronological age and age group) were used to assess possible sources of heterogeneity. Meta-regression was performed to determine whether chronological age (7 to 17 years) and age group (childhood (chronological age: 7 to 11 yrs) and adolescence (chronological age: 12 to 17 yrs)) were influencing LLP.

The agreement values of the studies in relation to the STROBE criteria were indicated in percentiles. The relative value of 100% was taken into account for the total number of items on the chek list. Subsequently, the relative value of items in which the studies were in accordance with the chek in list STROBE was calculated. The Kappa reliability index was calculated among the evaluators, to verify the degree of reliability in relation to the criteria of the bias analysis scale. The magnitude used was: absent [34]: Kappa = ≤0; poor: Kappa = 0–0.19; weak: Kappa = 0.20–0.39; moderate: Kappa = 0.30–0.59; substantial: Kappa = 0.60–0.79; and almost complete: Kappa = ≥ 0.80. All analyses were performed using open source software R (version 4.0.1; Foundation for Statistical Computing®, Vienna, Austria) and alpha was set at p <0.05.

## 3. Results

### 3.1 Search for articles in the databases for the present study

Table 1 shows the number of articles found in each database according to the search criteria, by semester, during the data collection period. The first 4 semesters correspond to the first research strategy, and the last two semesters correspond to the data resulting from the adjusted research grid (See Section: Identification of Studies and Search Strategy). Thus, in the year 2019 and in the first stage of the year 2020 the searches took as the starting date the day of the previouse search. In the second half of 2020, searches considered the time interval identified in the search strategy (2010 to 2020), and in 2021 searches were made taking as the starting date the day of the previous search.

**Table 1:** Results of the number of articles selected by search steps.

Sources	2^nd^ semester 2018	1^st^ semester 2019	2^nd^ semester 2019	1^st^ semester 2020	2^nd^ semester 2020	1^st^ semester 2021
SportDiscus	234	05	23	87	27.468	190
Cinahl	382	01	06	27	106.696	1.260
Scopus	06	0	0	0	0	0
PubMed	0	0	0	1	755.141	3.768
Google Scholar	6.990	210	8	176	16.800	361

### 3.2 Agreement among the researchers regarding the screening of studies

**Fig 1:**
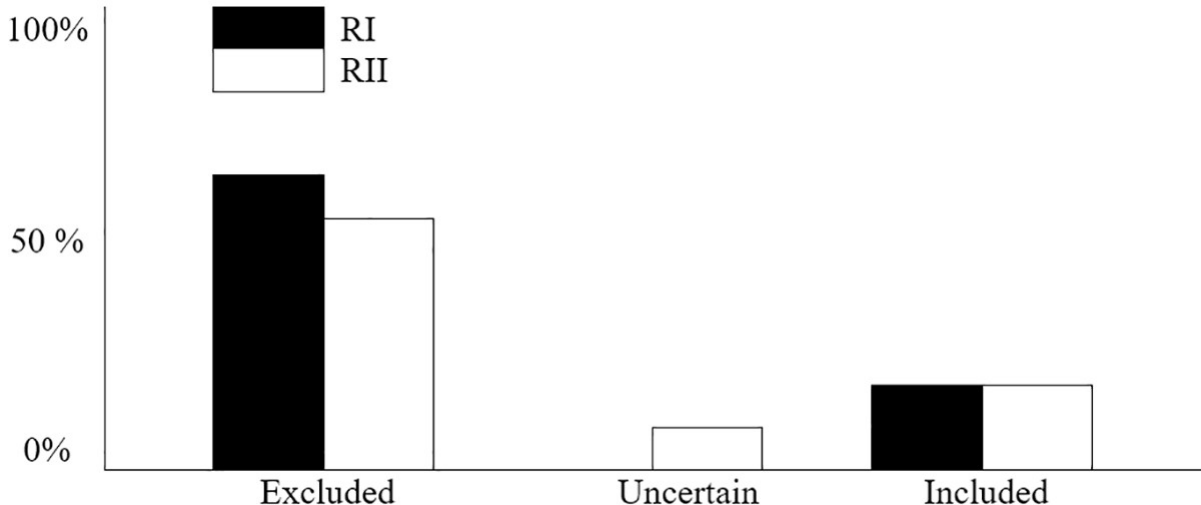
Agreement between the authors during the screening carried out via Rayyan. RI: Researcher I. RII: Researcher II.

Fig 1 depicts the graphs of agreement regarding the screening of studies included in the Rayyan online tool, both researchers arrived at the same results in relation to the inclusion of articles (25.1%). However, when dealing with exclusions, researcher I (P.F.A-N) excluded 74.9% of the articles while researcher II (V.M.M.O) excluded 73.2% of the data and was undecided in 1.7% of the analysed texts (i.e., undecided as to inclusion). In addition, the inter-rater agreement was almost complete (Kappa = 0.835; p = 0.04).

### 3.3 Reasons for inclusion and exclusion of studies in the present review

**Fig 2:**
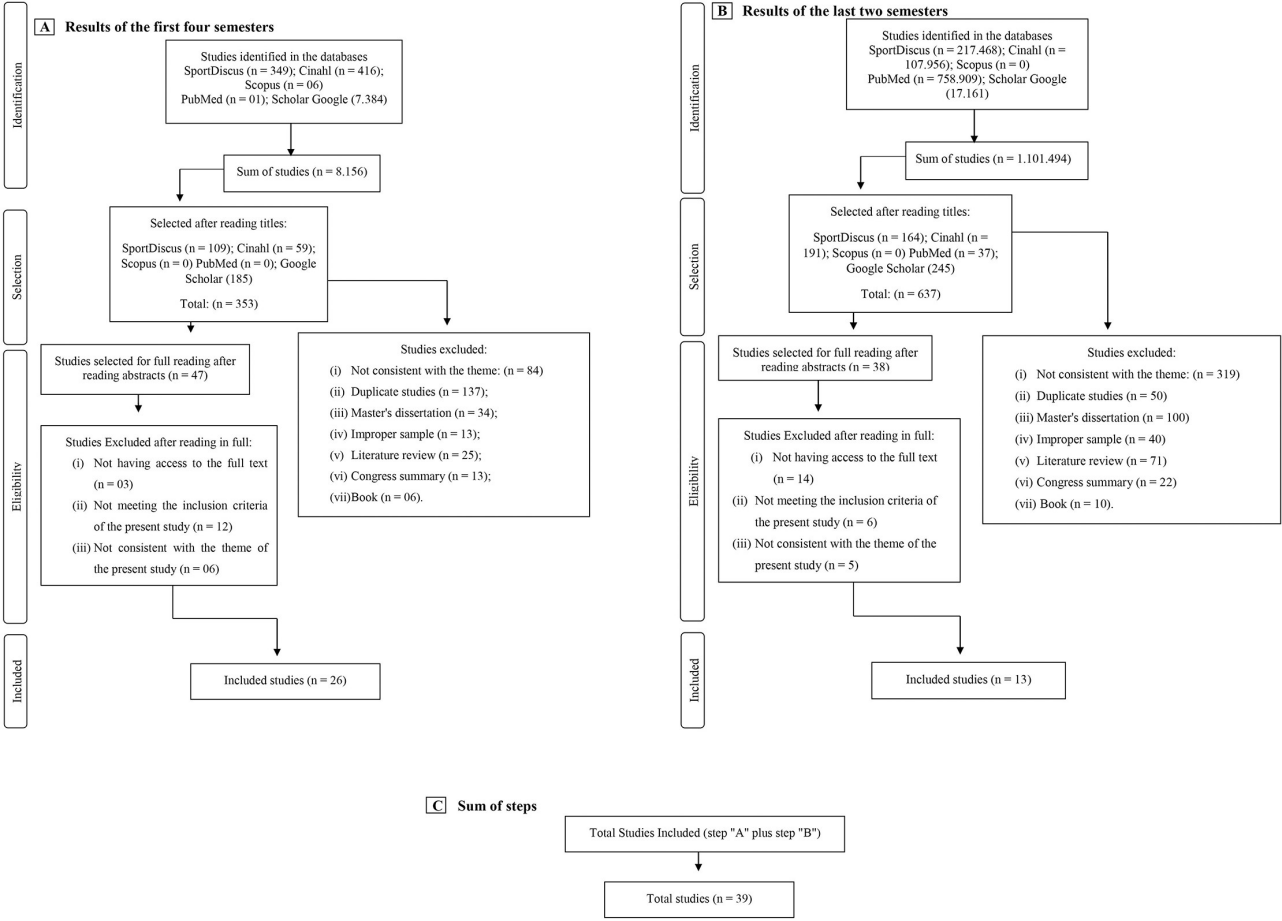
Flowchart of identification and selection of articles for systematic review.

Fig 2 (part “A”, “B” and “C”) shows the flowchart of the general outline of the present study in relation to the identification and selection of the texts included in this systematic review. The flowchart shows the total number of texts found, the number of articles included and excluded, and the reason for exclusion. The results of Fig 2A, refer to the first four semesters of the bibliographic searches (See Section: Identification of Studies and Search Strategy). While the results of Fig 2B, refer to the last two semesters of the bibliographic searches (See Section: Identification of Studies and Search Strategy).

### 3.4 Characterization of the studies included in the present review

In relation to the main outcomes of the studies conclusions included in this systematic review, the level of physical activity is identified as a determining factor for LLP in individuals of both sexes in 8% (n = 3) of the studies (see Table 2). In other words, subjects with a higher level of physical activity had higher LLP capacity. In addition, 18% (n = 8) of the articles indicated that advanced biological maturation significantly influenced the LLP of male subjects, while 3% (n = 1) of studies found a similar result in female subjects and 8% (n = 3) of the texts had similar conclusions for subjects of both sexes. However, another 6% (n = 2) of authors stated that, for females, biological maturation was not a determining factor for LLP.

**Table 2:** Characteristics of the studies.

References	Sample	Methodology	Outcome
Mariano et al. [35]	• 21 male football players.	• LLP tests: Squat Jump and Countermovement Jump. Continuous vertical jumps for 5 seconds.	There was significant variance in the continuous vertical jump test for 5 seconds in relation to puberty. Thus, the process of increasing LLP in pubertal subjects is greater than in post-pubertal.
		• Analysis of body composition.	
	• Age: 14 to 17 years.	• Analysis of puberty (Tanner).	
Da Silva et al. [36]	• 120 male teenagers.	• LLP test: Horizontal jump.	LLP in G2 and G3 was significantly higher among subjects with normal and late maturation stages. Thus LLP is influenced by puberty, in individuals in more advanced stages and with a chronological age above 12 years.
	• Age: 10 to 15 years.		
	• Groups: (G1-10 to 11 years), (G2-12 to 13 years), (G1-14 to 15 years).	• Analysis of puberty (X-ray of hand and wrist)	
Silva and Oliveira [19]	• 128 female adolescents.	• LLP test: Vertical thrust.	There was no difference in the strength of lower limbs between the groups (non-matured and matured). Thus, maturation did not influence the LLP of female adolescents.
	• Age: between 11 and 14 years old.		
	• Groups: non-matured (n = 72) and matured (n = 56).	• Analysis of puberty (menarche).	
Luguetti, Té and Boheme [5]	• 3,145 schoolchildren (1590 boys and 1555 girls).	• LLP test: Horizontal jump.	Male subjects had superior LLP compared to female subjects, regardless of age group. In addition, advanced chronological age was associated with increased LLP in males.
	• Age: between 7 and 16 years.		
Chillón et al. [37]	• 2,569 school children (1,068 children (550 males and 518 females), e 1.501 adolescents (775 males and 726 females).	• LLP test by Standing long jump.	In general, young people living in rural environments had better physical fitness than young people living in urban environments. However, for LLP, the environment in which they live did not show any significant associations.
	Age: children from 7 to 12 years old, and teenagers from 13 to 16 years old.	• Check if the environment in which you live (Urban vs. Rural) was associated with physical fitness.	
Rover et al. [20]	• Schoolchildren of both sexes.	• LLP test: Horizontal jump.	Girls and boys active in extracurricular exercise programs showed better LLP when compared to their peers.
	• Age: between 11 and 14 years old.		
	• Groups: practitioners and non-practitioners of extracurricular exercise programs.		
Carvalho et al. [38]	• 55 male basketball players.	• LLP test: Analyzed the isokinetic strength of the knee joint using a dynamometer.	Puberty was associated with knee extensors concentric (r = 0.64, p<0.001), knee extensors eccentric (r = 0.55, p<0.001), knee flexos concentric (r = 0.63, p<0.001) and knee flexors eccentric (r = 0.61, 0<0.001). Suggesting that the individuality of the subjects needs to be considered for sports training
		• Analyzed puberty by peak growth rate.	
	• Age: 15.1 ± 0.47 years.	• Verified the relationship between puberty and the strength of the knee joint	
Aouichaoui et al. [22]	• 391 young athletes (208 boys and 183 girls).	• LLP test: squat jump and countermovement jump.	Chronological age, body weight, height and fat free weight were indicated as predictors of LLP performance.
	• Age: between 7 and 13 years.	• Anthropometric analyses.	
Da Silva et al. [39]	• 11 male children.	• LLP test: vertical jump (intermitents for 30-s).	Young people inserted in a modality that allowed more sports stimuli showed superiority in LLP, compared to those inserted in a specific modality such as soccer.
	• Age: between 09 and 12 years		
	• Groups: Sport Initiation Group (SIG) and Indoor Soccer (IS).		
Hoshikawa et al. [40]	• 24 soccer players and 11 subjects from the control group, both male.	• LLP test: Analyzed the dynamic force during knee extension and flexion at 1.05 rad / s with a dynamometer.	After 6 months, participation in competitive football training did not increase LLP.
	• Age: 12 and 13 years old	• Evaluated the sample at baseline moments and after 6 months.	
Quatman-Yates et al. [41]	• 39 female soccer athletes.	• LLP test by isokinetic dynamometer.	With the advancement of puberty, the knee extension strength increased (p <0.05). The strength of the hamstring / quadriceps ratio decreased from pre-pubertal to pubertal stages (p <0.05). Suggesting, imbalances between the hamstrings and the strength of the quadriceps that seemed to arise with the advancement of puberty.
		• 12 months of follow-up, carrying out evaluations every four months.	
	Age: 11 to 13 years	• Analysis of puberty (sexual maturation).	
Silva et al. [42]	• 38 adolescents (24 females and 14 males).	• LLP test: Horizontal jump	LLP correlated with the% fat (r = 0.24; p = 0.03). LLP correlated with supraspinal skinfolds (r = −0.45; p = 0.00); tricipital (r = −0.23; p = 0.03) and subscapular (r = −0.30; p = 0.00). Thus, higher LLP was associated with lower adiposity.
	• Age: 15.9 ± 0.81.	• Analysis of body composition.	
Filho [43]	• 24 Schoolchildren.	• LLP test: Horizontal jump analysed in the conditions before and after the intervention of weeks.	Intragroup LLP did not change significantly (p> 0.05). Indoor soccer group were superiore compared to the handball group, in the pre- and post- intervention conditions in relation to LLP.
	• Age: between 11 and 13 years old.		
	• Groups: Indoor Soccer and Handball.	• 12 weeks of training and sports gestures related to futsal and handball sports.	
Tounsi et al. [25]	• 525 Tunisian teenagers (242 male and 283 female).	• LLP test: squat jump and countermovement jump.	Age, weight, trunk height, waist circumference, fat-free mass and leg muscle volume were significant predictor of LLP in boys. Body weight was the best predictor of LLP in girls.
	• Age: between 13 and 17 years old.	• Anthropometric and morphological analysis.	
Martins et al. [21]	• 46 male children	• LLP test: squat jump.	Children participating in sports initiation were superior in relation to the biomechanical parameters of LLP. (p <0.05).
	• Age: 10.4 ± 1.08 years.		
	• Groups: School physical education (Control Group) and Sports Initiation (Experimental Group).		
Hoffmann et al. [44]	• 20 male adolescents, participants in systematic indoor soccer training.	• LLP test: Horizontal jump.	Advanced puberty was associated with height and better LLP performance (p <0.05). Young people with lower body adiposity were superior in LLP (p <0.05). Young people with higher stature were superior in LLP in relation to the lowest (p <0.05).
	• Age: between 14 and 15 years.	• Anthropometric and body composition analysis.	
		• Analysis of Puberty (Tanner).	
Silva et al. [45]	• 12 male adolescents, soccer players.	• LLP test: Vertical jump	The player’s position on the football field did not influence LLP. (p <0.05).
	• Age: 14.7 ± 1.2 years.		
	• Groups: side and midfielders.	• Test flexibility: Posterior chain of the lower limbs.	
Mello et al. [11]	• Brazilians of both sexes (Males: 4,751, Females: 3,904).	• LLP test: Horizontal jump.	40.2% of male subjects indicated LLP to be "weak" and 3.7% indicated LLP to be "excellent". 43.7% of female subjects indicated weak LLP and 4.2% indicated “excellent” LLP.
	• Age: between 7 and 17 years.		
Rezende et al. [46]	• 122 schoolchildren of both sexes.	• LLP test: squat jump and countermovement jump.	Only subjects in the sixth year showed significant results in the countermovement jump test. Being the highest values obtained by students of a private school (p = 0.018).
	• Age: between 10 and 15 years.		
	• Groups: public school and private school.		
Gantois et al. [47]	• 239 subjects of both sexes;	• LLP test: vertical jump.	Young people with delayed maturation were lower in LLP compared to those with advanced maturation (p <0.05). 36% and 19.2% of the LLP variance was shared by the bone age of boys and girls (45.2% and 16.1% respectively).
	• Age: between 10 to 13 years.	• Analysis of puberty (bone age predictive equation).	
Barros et al. [48]	• 133 female adolescents	• LLP test: Sargent Jump.	The maturational advance of adolescents did not influence LLP.
	• Age: between 10 and 17 years old.	• Analysis of puberty (Tanner).	
Feitoza, Camara and Gomes [49]	• 84 students (57.1% female and 42.8% male).	• LLP test: Horizontal jump.	The LLP of the investigated students were predominantly located in the “weak” level, in both sexes.
	• Age: between 7 and 10 years.		
Pinto et al. [24]	• 89 adolescents of both sexes.	• LLP test: vertical jump.	The levels of maturation, testosterone and estradiol played an important role in the physical aspects and in the performance of motor skils of LLP in adolescents (both sex).
	• Age: between 10 and 14 years.	• Analysis of puberty (bone age predictive equation).	
		• Testosterone and estradiol analysis.	
McKinlay et al. [50]	• 41 male soccer players.	• LLP analyzed by countermovement jump, squats, explosive knee extensions, unilateral and dynamic isometric (240° / s) (Biodex System III).	Peak torque (pT) and peak torque development rate (pRDT) were not related to LLP (p> 0.05). Dynamic pT and pRTD, normalized for body mass, were related to LLP in all tests (r = 0.38–0.66, p <0.05).
	• Age 12.5 ± 0.5 years.		
Agresta et al. [51]	• 45 healthy young people (24 females and 21 males).	• They filmed the execution of 10 single leg squats for each leg.	There was no difference in the performance of LLP between the different stages of puberty. Chronological age was associated with LLP in both sexes (p = 0.02).
	• Age: 8 and 17 years old (13.3 ± 3.27).	• They analyzed puberty by the peak of heigth velocity.	
Moreira et al. [52]	• 22 males teenagers.	• LLP test: Horizontal jump.	The performance of the lower limbs was as expected, higher in subjects with a higher chronological age (p <0.05).
	• Age: 14.1 ± 1.44 years.		
	• Groups: group: 11–14 years and group: 15–16 years.		
Aouichaoui et al. [23]	• 1,055 Tunisian athletic children and adolescents (643 boys and 412 girls).	• LLP test: squat jump and countermovement jump.	Between 12 and 18 years of age, male subjects were superior to females in LLP (p <0.05). The girls’ mean LLP power was higher between 13 and 16 years old (p <0.05).
	• Age: between 7 and 17 years.	• Anthropometric and morphological analysis.	
Dantas et al. [53]	• 76 teen rowing athletes (37 boys and 38 girls).	• LLP test: Horizontal jump.	Boys (p = 0.027) and girls (p <0.001) with advanced puberty had higher LLP compared to their peers with late puberty. Thus, puberty was related to LLP (r> 0.7; p <0.05).
	• Age: between 8 and 14 years.	• Analysis of puberty (bone age predictive equation).	
Weedon et al. [16]	• 718 participants (311 girls, 407 boys).	• LLP test by broad jump.	LLP motor competence was related to BMI (females r = -0.29, p<0.05; males: r = -0.17, p<0.05; combined sexes: r = -0.25, p<0.05).
	• Age: 13 to 14 years.	• Analysis of the body mass index (BMI).	
Dobrowoski et al. [54]	• 74 male students attending a futsal sports initiation program.	• LLP test: Horizontal jump.	Subjects in advanced maturation stage were superior in LLP (p <0.05).
	• Age: between 11 and 15 years.	• Analysis of puberty (Tanner).	
Pinto, Araújo and Filho [55]	• 32 male athletes from the Under-14 and Under-15 categories.	• LLP test: Counter Movement Jump.	The differences between the modalities do not seem to be significant for LLP. (p <0.05).
	• Age: between 14 and 15 years.	• Anthropometric and body composition analysis.	
	• Groups: Volleyball and Football.		
Almeida-Neto et al. [3]	• 44 female subjects	• LLP test: Countermovement jump.	LLP was related to hormonal markers and biological maturation (estradiol: r = 0.52; p = 0.006; testosterone: r = 0.42; p = 0.03; Somatic maturation: r = 0.59; p <0.0001; Bone maturation: r = 0.55; p <0.0001).
	• Age: 11.5 ± 1.50 years.	• Analysis of puberty (predictive equations for bone age and peak growth velocity).	
		• Testosterone and estradiol analysis.	
Xu et al. [56]	• 22.681 children and teenagers (11.300 males and 11.381 females)	• LLP test by standing board jump	Adolescents with adequate BMI showed better performance of LLP regardless of age (p <0.05). Subjects of both sexes with a BMI below or above the average showed worse results for LLP regardless of age (p <0.05).
	• Age: 10 to 17 years	• They verified the relationship between body mass index (BMI) and physical fitness.	
Zanini et al. [57]	• 44 male athletes from the under-12 and under-13 categories.	• LLP test: Vertical Jump.	The results showed a correlation of LLP (p = 0.003; r = -0.43) with the fat percentage.
	• Age: between 12 and 13 years.	• Analysis of body composition.	
Almeida-Neto et al. [4]	• 64 young athletes of both sexes (73% male and 27% female).	• LLP test by vertical jump and jump against movement	Athletes with advanced puberty showed higher concentrations of lean mass and superiorr LLP (p <0.05). Puberty interacted with lean mass (η^2^p = 0.753). Lean mass showed a local effect (ƒ^2^) higher than that of puberty for LSS.
		• Puberty analyzed by the peak growth rate.	
	• 13,6 ± 1,17 years.	• Lean mass analyzed by dual energy X-ray absorptometry (DXA).	
		• Controlled the covariate sex by ANCOVA test.	
Mandroukas et al. [58]	• 126 soccer athletes and 60 untrained subjects (control group), all male.	• LLP Analyzed by dynamometer: peak values of isokinetic-concentric torque of the hamstrings (H) and quadriceps (Q), as well as the conventional strength relationships of H: Q and angular velocities of 60, 180 and 300° ·s^-1^	The absolute isokinetic-concentric muscle strength was significantly higher (p <0.001) in the soccer group aged 12 and 16, compared to the control group, for knee flexors and extensors. No significant differences were found between soccer players and the 14-year-old control group for the muscle groups of Q and H.
	• Age: 12 to 16 years	• Analyzed whether soccer training was associated with LLP.	
Itoh; Hirose [59]	• 49 elite male soccer players.	• LLP test by Five-step jumped.	For LLP, the groups with early and average puberty showed better results than the group with late puberty (η^2^p = 0.24; p <0.05).
	• Age: 12.7 ± 0.2 years.	• Analysis of puberty by X-ray of the hand and wrist.	
		• Sample segregated at puberty: early, medium and late.	
Almeida-Neto et al. [13]	• 37 male athletes.	• LLP test: Squat jump.	Sexual maturation was related to LLP (r = 0.61, p <0.0001). Young people with advanced sexual maturity showed superiorite LLP in relation to those with late sexual maturation.
	• Age: 11.3 ± 0.94 years.	• Analysis of puberty (predictive equation for sex maturation).	
Guimarães et al. [60]	• 160 male basketball players.	• LLP test by squat jump and countermovement jump	The individual differences that occured during puberty influenced the development of LLP in adolescent basketball athletes. In addition, the increase in LLP was related to the increase in body weight.
	• Age: 11 to 15 years.		
		• Longitudinal follow-up of 3 years, evaluations took place at intervals of 6 months.	
		• Analysis of puberty by peak heigth velocity.	

It was observed that 3% (n = 1) of the articles affirm that puberty and the levels of steroid hormones were determinant for LLP in female individuals and 3% (n = 1) of papers indicated similar result in male athletes. In contrast 3% (n = 1) of studies indicated that only steroid hormones were determinants for LLP in individuals of both sexes. Regarding the biomechanical experience in performing jumps (task specificity) for the use of the LLP, 8% (n = 3) of the studies found that it is a significant factor, while another 8% (n = 3) indicated that it is an irrelevant factor for LLP. Anthropometric characteristics (8% of texts (n = 3)), sex (6% of texts (n = 2)), advanced chronological age (12% of texts (n = 6)) and body morphology (6% of texts (n = 2)) were also identified as factors associated with higher levels of LLP (See Table 2).

### 3.5 Data groupings

**Fig 3:**
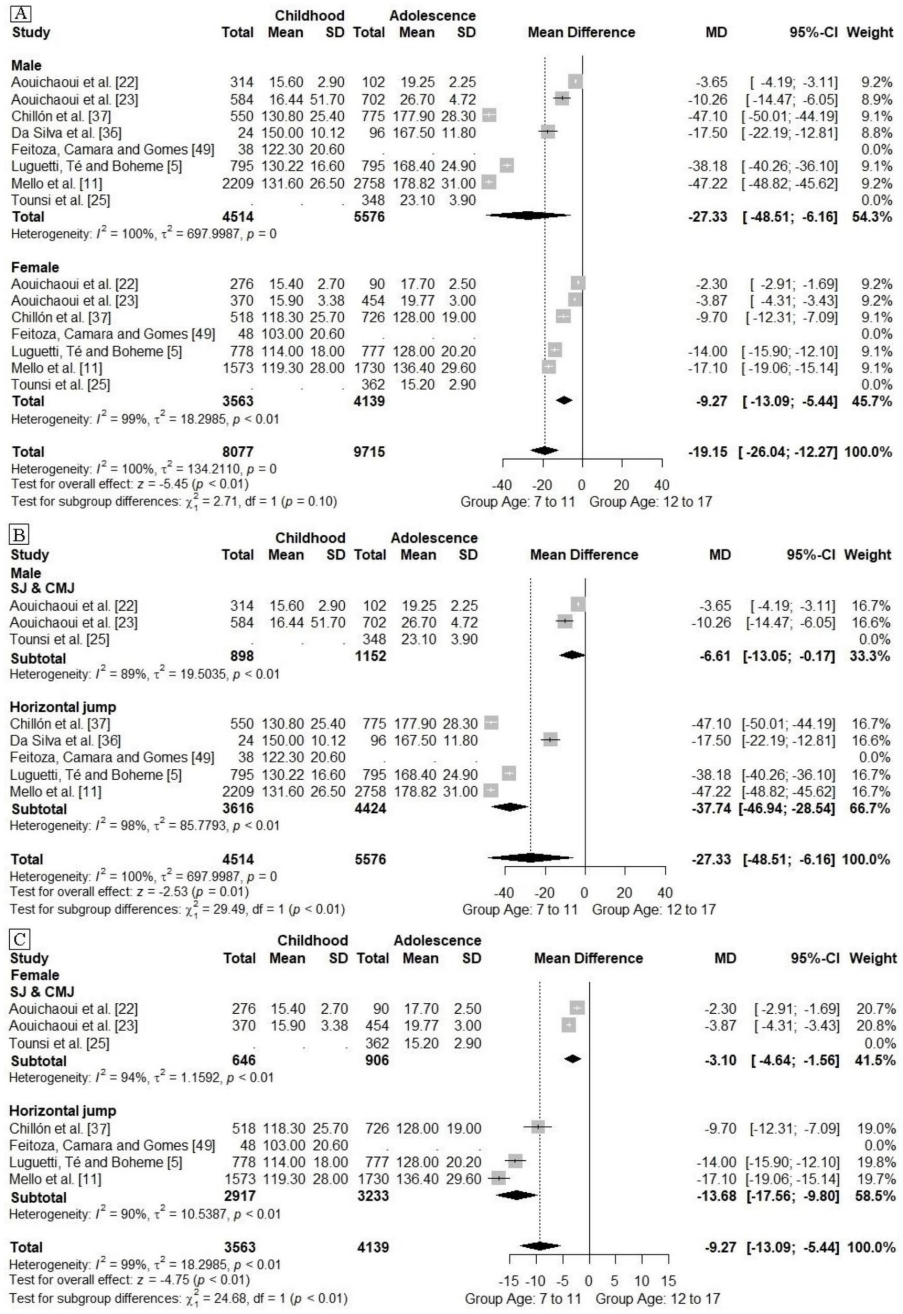
Meta-analysis grouping the studies by age group, sex, and by lower limbs performance analysis protocol. SJ:Squat jump. CMJ: Countermovement jump. SD: Standard deviation. MD: Mean Difference.

In Fig 3 studies are grouped dependant on available means and standard deviations data of LLP by sex and age group. Thus, two groups were formed: Childhood (formed from data from subjects aged 7 to 11 years) and adolescence (formed from data from subjects aged 12 to 17 years). It was found that, with respect to the overall effect, the adolescence group had higher LLP when compared to the childhood group (Fig 3A). This was verified by subgroup analyses which showed that this occured regardless of sex and the jumping protocol used to analyze LLP (Fig 3B and 3C). However, the data grouped in Fig 3 shows a very high heterogeneity, indicating that although the overall effect favors the adolescent group, there are perculiarities in the studies that require caution when analyzing this finding.

Given the heterogeneity of the results found in the data grouping in Fig 3, a meta-regression analyses was performed to explain the reasons for the wide heterogeneity among the studies grouped by age group. Thus, it was indicated the high heterogeneity among the studies could be accounted for by the variety of sample sizes of the surveys (which ranged from 24 to 2758 subjects) and the variety of chronological age of the samples grouped by age groupings, which ranged from 7 to 11 years for childhood and from 12 to 17 years for adolescence (See Table 3).

**Table 3:** Meta-regression analyses to justify the heterogeneity found among the studies in the meta-analyses regarding the different age groups (childhood and adolescence).

Meta-Regression / Random Effect	Point Estimate	Standard error	CI 95%	Z	p
	Male Sample				
Intercept	-10.4	0.7	-11.8; -9.0	-14.9	<0.001
Sample Size	0.3	0.01	0.2; 0.3	379.7	<0.001
Grouped age (Childhood and Adolescence)	2.5	0.3	1.9; 3.0	8.5	<0.001
	Female Sample				
Intercept	8.3	0.5	7.4; 9.2	17.7	<0.001
Sample Size	0.24	0.01	0.23; 0.25	484.2	<0.001
Grouped age (Childhood and Adolescence)	-1.4	0.2	-1.8; -1.0	-6.9	<0.001

**Fig 4:**
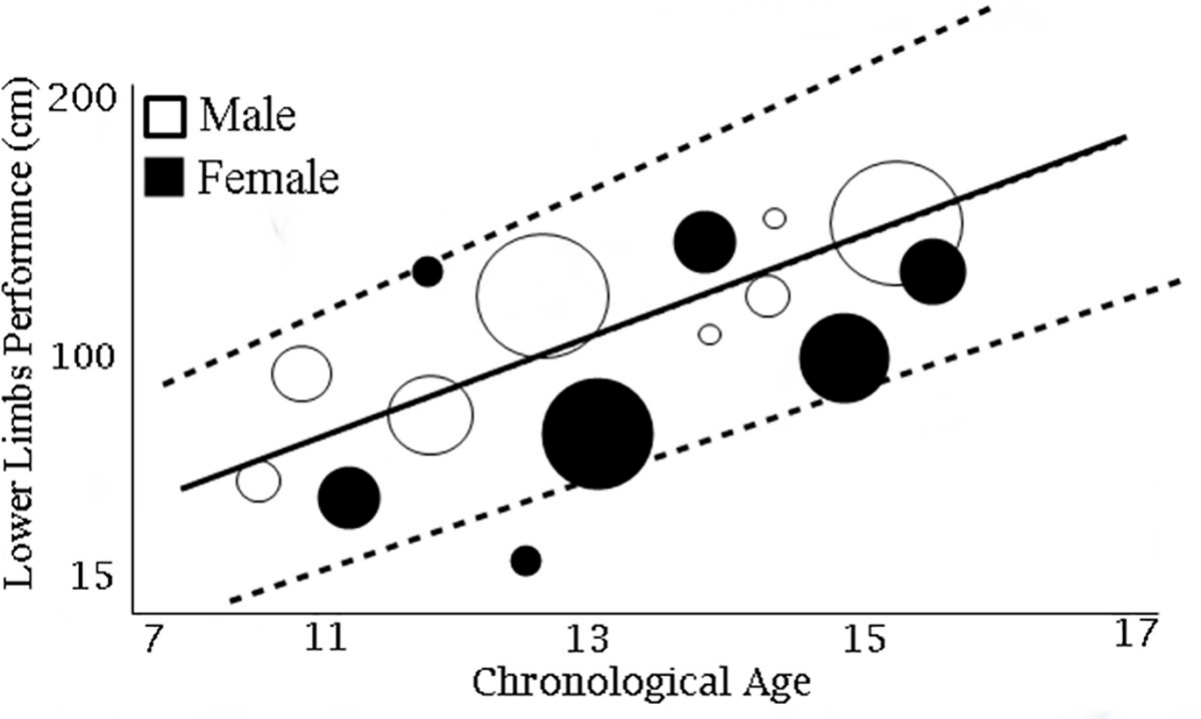
Meta-regression analyses to verify the relationship of chronological age with lower limbs performance. (cm): Centimeters. —-: Confidence Interval 95%.

In addition, Fig 4 displays the metaregression analysis that encompassed the studies that compared LLP in different chronological age groups (ranging from 7 to 17 years), indicating that chronological age was related to increased LLP in males (Point estimate: 1. 9; Standard error: 0.04, 95% CI: 1.7; 2.0; Z: 39.1; p<0.001) and females (Point estimate: 0.3; Standard error: 0.03, 95% CI: 0.3; 0.4; Z: 10.1; p<0.001), and that chronological age explained the heterogeneity among the studies grouped in Fig 3 by 70% for males and 68% for females.

**Fig 5:**
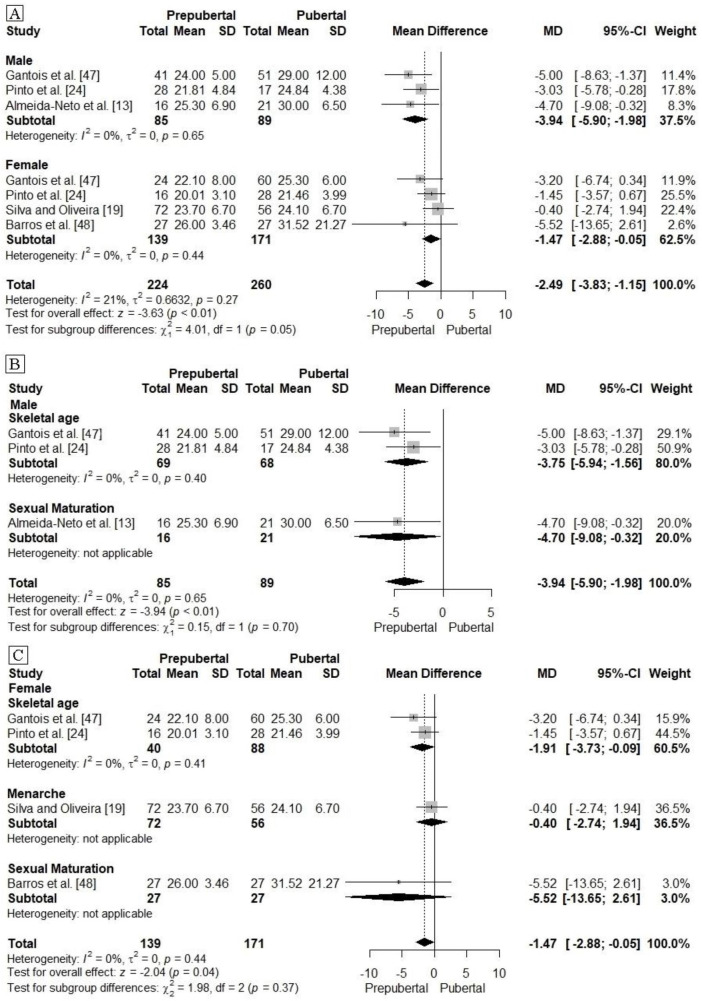
Meta-analysis grouping the studies by stage of biological maturation [prepubertal and pubertal]. SJ:Squat jump. CMJ: Countermovement jump. SD: Standard deviation. MD: Mean Difference.

In Fig 5 studies were grouped by those studies which had means and standard deviations of LLP when analysing by stage of biological maturation (pre-pubertal and pubertal). Thus, the overall effect of the analyses indicated that subjects during puberty had superior LLP when compared to their pre-pubertal peers (Fig 5A). However, subgroup analyses indicated that for male subjects the result remained similar to the global one identified (Fig 5B), while for female subjects there was no significant difference between the subgroups (Fig 5C). For the aforementioned analyses, the levels of heterogeneity between the studies were acceptable (<25%).

### 3.6 Study quality and basic requirements for an observational study

**Fig 6:**
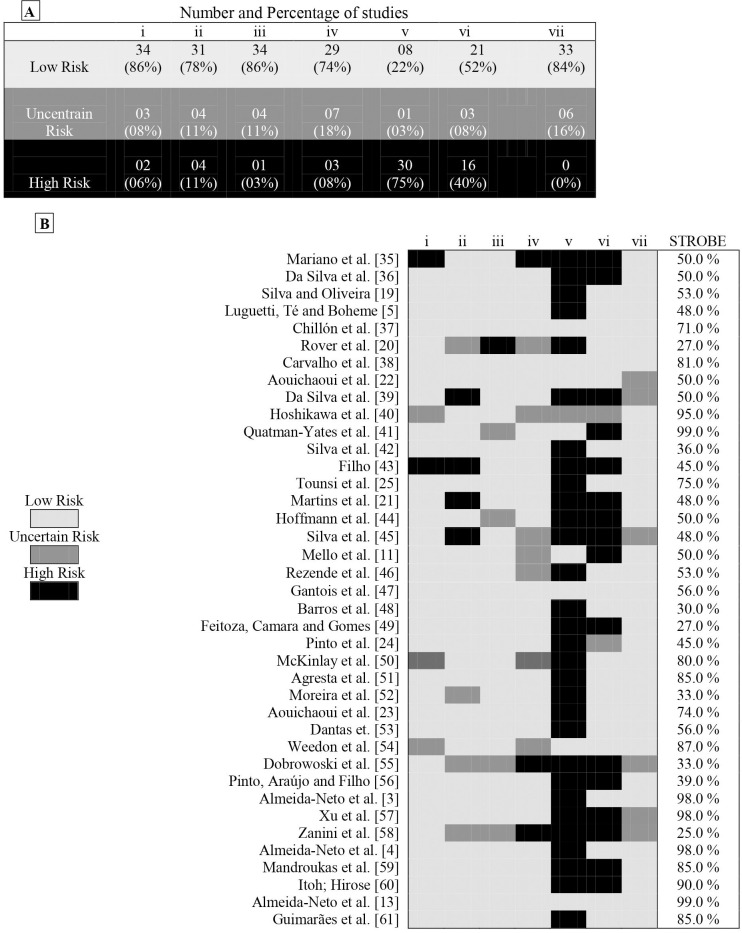
Graphical representation of bias analysis and of analysis of the components of the chekin linst STROBE. i: The study does not answer the research question adequately. ii: The sample base and the sample size were not adequate. iii: Objective, adequate and standardized criteria for the outcome were not used. iv: The outcome was measured in a skewed manner. v: Results are not given with a confidence interval when appropriate. vi: The study does not expose practical applicability. vii: The study has no reproducibility for other samples from different populations. STROBE—Percentage of minimum requirements met for observational studies.

Fig 6 reports the analysis of biases of the analysed studies. In general (Fig 6A) and individually (Fig 6B), studies presented a high risk for not presenting confidence intervals appropriate for their studies. Thus, 10% of studies indicate d high methodological quality (i.e., low risk in all criteria of the Loney scale [31]), while 59% displayed moderate methodological quality (i.e., high risk or uncertain risk in one or two items of the Loney scale [31]), and 31% indicated low methodological quality (i.e., high risk or uncertain risk in three or more items on the Loney scale [31]). When analyzing each item in the Loney [31], 80% of the analyzed articles indicated low risk of bias for subjects related to the sample, the outcome and the reproducibility of the research. However, 80% of the studies did not present the confidence intervals in the statistical analysis and about 46% of the authors did not indicate practical applicability for the study results.

In addition, the percent agreement of the studies with the STROBE requirements for observational studies are shown in the figures, which was used to help interpret the basic methodological requirements of each study included in this systematic review. Thus, four studies showed agreement between 27 and 30%, four articles showed agreement between 33 and 39%, five studies showed agreement between 45 and 48%, ten studies showed agreement between 50 and 56%, three studies showed agreement above 70%, six studies agreement between 80 and 87% and six studies showed agreement above 90% with the STROBE requirements (Fig 3B). It is noteworthy that the Kappa coefficient reliability index among the three evaluators was ≥0.723 (p = 0.02), which indicates good compatibility in relation to the bias analysis and in relation to the STROBE analysis.

Regarding the most frequently omitted STROBE by the studies, failure to explain the study hypothesis, this occurred in ~90% of the articles, ~86% of the studies did not state the research design in the manuscript title, ~75% of the studies omitted the sample recruitment process, ~65% did not report whether confounding factors were controlled for during study procedures, and ~88% did not address possible efforts to minimize biases during the research.

## 4. Discussion

The objective of this systematic review was to analyse factors associated with performance of lower limbs (LLP) in young boys and girls aged between 7 and 17 years.

The main findings indicated that advancing chronological age related to increased LLP, and that pubertal youth demonstrated higher LLP than their pre-pubertal peers. In addition, among the individual results of the studies gathered in the present review, it was found that body morphology was related to LLP. In this sense, our findings suggest that as chronological age advances (childhood to adolescence) a maturation of biological systems occurs and that consequently the phenomena triggered (i.e., growth spurt, increase in androgenic hormones, increase in lean mass levels, etc.) by puberty was associated with LLP.

When we grouped the studies by age group the results suggested that the transition between childhood and adolescence is related to LLP, where adolescents have superior values to their peers who are in childhood. However, the grouping of data pointed out a high heterogeneity (I^2^>50%), to try to identify the tail of the heterogeneity we performed subgroup analyses considering the jumping protocols used to analyze LLP, however the heterogeneity was reduced by less than 10% in the male and female groups, still being above 50%. Thus, the different jumping protocols used to analyze LLP do not seem to have influenced the high heterogeneity among the studies.

When performing meta-regression analyses to identify the factors that might be associated with high heterogeneity, it was found that variability in sample size among the pooled studies (which ranged from 24 to 2758 subjects) and variability in chronological age in the Childhood (from 7 to 11 years) and Adolescence (from 12 to 17 years) groups were associated with high heterogeneity. Through a new meta-regression we identified that advancing chronological age was associated with increased LLP, and that this association explained the heterogeneity across studies by 70% for males and 68% for females.

In this sense, based on the present systematic review when disregarding sex (male and female) the stage of puberty may have beeen a factor associated with LLP. Thus, for both sexes, pubescent subjects showed higher levels of LLP than prepubescent subjects. However, when considering the male and female subgroups for protocol for the analysis of biological maturation, it was found that male pubertal subjects perform better in LLP than their prepubertal peers, regardless of the protocol used (i.e., skeletal age and sexual maturation). Howere no differences were found in females subgroups (i.e., skeletal age, sexual maturation and menarche). This can be explained by the fact that peak biological maturity in female subjects occured about two to three years earlier compared to their male peers [18, 61, 62]. In addition, there were a low number of studies in each analyzed subgroup, it is suggested that by increasing the sample size in the subgroups, the results could have pointed to significant differences.

The heterogeneity, although among the studies was acceptable, we must consider that the grouped studies used different indirect protocols (i.e., equations, age at menarche and secondary sexual characteristics) to identify the stages of puberty. When assessing puberty by indirect methods, at the end of the analyses the tools generate numerical scores that group subjects into puberty levels that can vary in three stages for skeletal age (1: late or pre-pubertal. 2: average or pubertal. 3: early or post-pubertal), in two stages for age at menarche (1: pre-pubertal. 2: pubertal), and in up to 5 stages for secondary sexual characteristics (1: pre-pubertal. 2 to 4: pubertal. 5: post-pubertal) [3, 13, 18, 19]. This variety in methods of classification may have influenced the results of the present study.

When considering articles that were not part of the meta-analysis, due to the non-information of the central tendency measures of the maturational groups, this variability in the estimation of pubertal status contradicts the findings, demonstrating the absence of a consensus on the relationship of puberty with LLP [3, 19, 24, 35, 36, 40, 47, 48, 53, 54]. We emphasize that one must also consider confounding factors related to the puberty process that may also influence or relate to LLP, such as body morphology for example.

It is known that with the advancement of puberty there will increase the physiological stimuli that promote the secretion of androgenic hormones such as testosterone and estradiol [3, 18, 24, 63]. In subjects of both sexes the increased levels of hormones may favor lean mass gain [64, 65]. However, there is a strong interaction of adequate nutrition and the level of physical training in relation to reaching the apex this is favoring [10].

In this context, the level of physical activity and time of exercise can also provide the subjects with a greater biomechanical experience in performing jumps, and other specific movements of sports that have the predominance of the lower limbs such as running and kicking, for example [66]. Thus, according to the review carried out by the present study (See Table 2), it was possible to suggest that the mastery of the technique (i.e., biomechanical factors linked to the vertical jump or the counter-movement jump) was associated with the specific training of the sport to which the subjects were part of (i.e., volleybal, basketball, soccer, etc.) as well as the level of physical activity, [21, 37, 39, 41–42, 45, 49].

Regarding the quality of the articles contained in the present review, the sampling power and reproducibility indicated low levels of bias (See Fig 6B), suggesting that the outcomes of the research results contained in this review were appropriate. However, most of the studies analysed by the present review did not present the confidence intervals (CI) of their findings. It is known that CIs are used to indicate the reliability of an estimate [67]. Thus, among similar estimates, those that indicate a narrowe CI indicate more reliable results in relation to those with a wider CI [67]. Moreover, 36% of the articles brought together in this review, did not indicate a practical applicability for their results. Research without practical applicability does not offer objectivity, which can make it unfeasible for the professional field and unattractive for readers [68].

Within this perspective, the analyses of the present study also indicated that a significant amount of studies included in this review (59%, see Fig 6B), presented less than 70% of the essential items for an observational study, and this may suggest caution when interpreting the findings identified. Methodological flaws can be easily committed when the requirements involved in the design of a research study are not understood, which can skew the results to inadequate estimates [32, 69–71].

The present study has among its strengths the fact that the methodology has been turned to the analysis of observational studies, which are seldome explored in systematic review articles. Identifying that there are several divergences in relation to the methodologies used in these studies, is useful in encouraging future studies to seek a higher methodological quality in relation to the criteria required for this type of scientific research. In addition, this review contributes to the work of health and sport professionals, in helping them explain what factors should be analysed and / or stimulated to favor the LLP in children and adolescents.

However, the present study has the following limitations: (i) The set of descriptors used in the methodology has a limited scope; (ii) Despite the relevance, the databases used may not have been enough for the collection of studies available on the subject.

## 5. Conclusion

It is concluded that the factors associated with lower limbs performance in youths of both sexes in the age group between 7 and 17 years old are still inconsistent in the literature. However, both advancing chronological age and stage of puberty are associated with increased lower limbs performance. In addition, the studies reviewed suggest that the level of physical activity, body morphology and hormonal factors are additional factors associated with the lower limbs performance.

## Supporting information

S1 Checklist (PDF)

S1 Graphical abstract (TIF)
