# Supplementary figures and images for: Factors related to lower limb performance in children and adolescents aged 7 to 17 years: A systematic review with meta-analysis
Source: PLoS One. 2021 Oct 6;16(10):e0258144. doi: 10.1371/journal.pone.0258144 (PMC8494314; doi:10.1371/journal.pone.0258144)

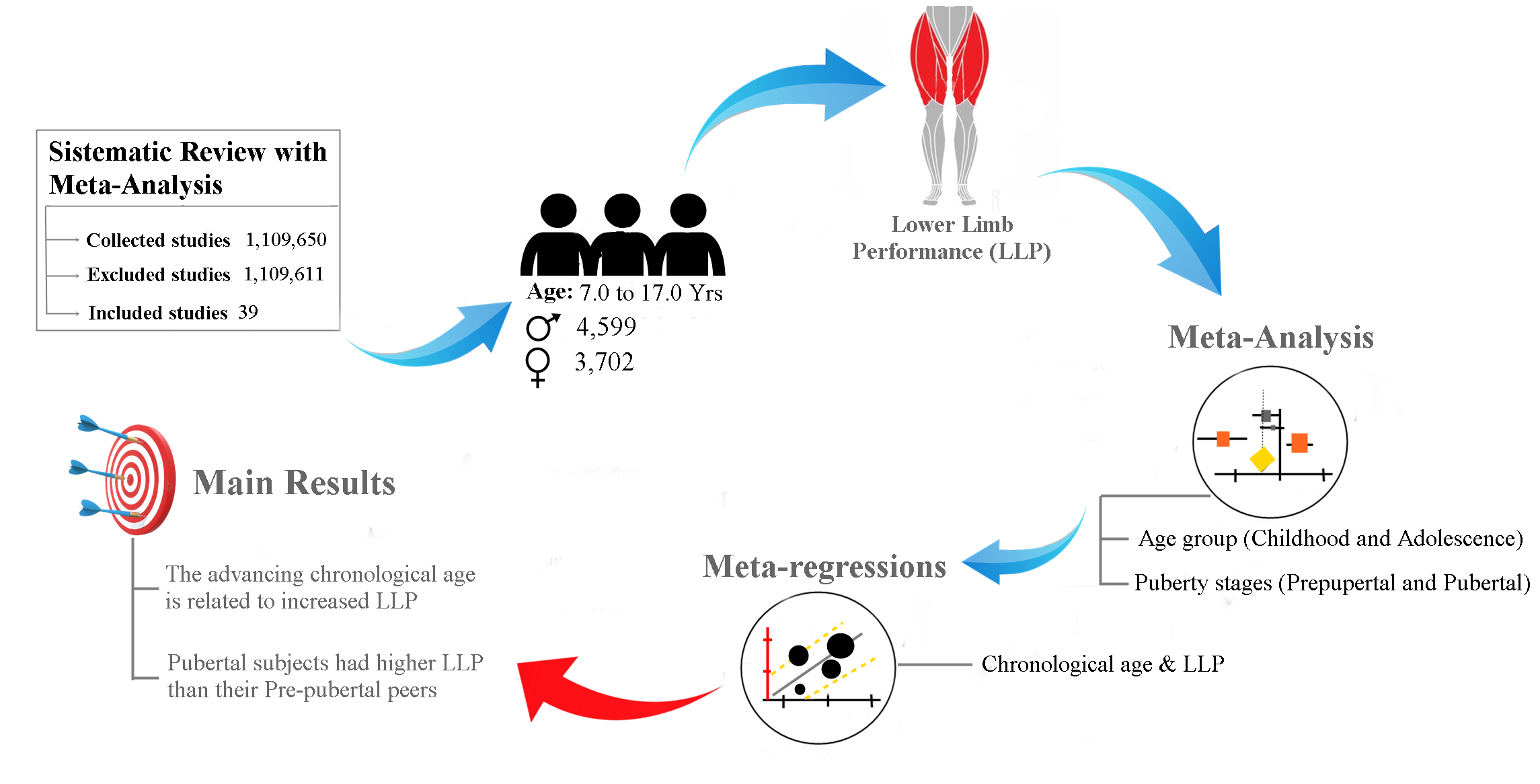

Supplement: S1 Graphical abstract — (TIF) [file pone.0258144.s002.tif]
